# The Application Value of Spiral CT Lung Densitometry Software in the Diagnosis of Radiation-Induced Lung Injury

**DOI:** 10.1155/2021/9305508

**Published:** 2021-12-20

**Authors:** Qingsong Tao, Ting Zhu, Xiaoqin Ge, Shengping Gong, Jianxin Guo

**Affiliations:** The Chemoradiotherapy Center, Ningbo No. 1 Hospital, Ningbo, Zhejiang 315000, China

## Abstract

In order to study the application value of spiral CT lung density measurement software in the diagnosis of radioactive lung injury, the average CT values of lung apex, hilum, and diaphragm were measured by Pulmo automatic evaluation software of 16-slice spiral CT in 96 patients with different types of radiation lung injury diagnosed by conventional CT and 80 healthy subjects. The radiation lung injury on CT slices was classified, and the lung density was measured. In 96 patients with different types of radiation lung injury, 56 patients had different degrees of increase in average lung density, which was most obvious in the type of air insufficiency and chronic fibrosis. CT values of lung density in the ground glass stage and patch stage of acute radiation pneumonia had little influence due to the range and time of exposure. The lung density of 35 patients with radiation injury was measured in the normal range. There was a significant difference between normal lung density and abnormal lung density in different types of radiation lung injury (*X*^2^ = 56.718, *P* < 0.001). The mean lung density of 68 cases was normal and that of 12 cases was abnormal. There was a significant difference in lung density between the lung injury group and the normal group (*X*^2^ = 18.027, *P* < 0.001). Spiral CT lung density measurement can accurately evaluate the lung density values of different types of radiation lung injury and judge the correlation between lung density and different types of radiation lung injury. It is of great value to diagnose, locate, and master the radiation dose of different types of radiation lung injury.

## 1. Introduction

Radiation-induced lung toxicity (RILT) includes early lung injury and late lung injury; early lung injury is also called acute radiation pneumonia, occuring within 3 months after the start of radiotherapy; radiation lung injury after 3 months of radiotherapy is called advanced radiation lung injury, and late injury generally refers to radiation pulmonary fibrosis. The cause of radiation lung injury is clear; it is mainly caused by a certain volume of lung tissue receiving a certain dose of ionizing radiation [1]. In the radiotherapy of lung cancer, esophageal cancer, and other chest tumors, after a certain volume of normal lung tissue is irradiated with a certain dose, damaging normal lung tissue, acute radiation pneumonia can occur when such radiation damage to the lung reaches a certain level, manifested as low-grade fever, cough, and chest tightness; severe cases can be manifested as dyspnea, chest pain, and persistent dry cough, if acute radiation pneumonia is not timely intervention and treatment; it can further progress to radiofibrosis of the lung, leading to severe damage to lung function and even death of patients [2]. Therefore, to study the mechanism of radiation-induced lung injury and how to effectively prevent it, treatment of radiation-induced lung injury has important clinical significance for the radiotherapy of thoracic tumors and the improvement of the quality of life of patients after radiotherapy [3]. Studies have shown that Fang et al. said that pulmonary interstitial lesions are pulmonary capillary vasculitis, when inflammation is limited to capillary endothelial cells; it can cause mild pulmonary interstitial changes, when epithelial cells are involved; it can cause acute alveolitis, when alveolitis is absorbed; it returns to chronic interstitial inflammation of the lung, if improperly treated or untreated; finally, it causes pulmonary fibrosis [4]. The exact mechanism of CTD with ILL is unknown, may be related to vasculitis, immune complexes in the blood vessel wall activate complement to release neutrophil chemokines, causes neutrophils to gather locally to release collagenase and free radicals, destroys the lung parenchyma, and causes alveolitis [5]. As the disease progresses, collagen is deposited extensively, leading to irreversible pulmonary interstitial fibrosis. Jayakrishnan et al. believed that KPS score <80 points is an independent risk factor for radiation-induced lung injury in patients with esophageal cancer IMRT (*P* < 0.05); it shows that physical fitness is an important factor affecting the occurrence of radiation pneumonia. The inconsistency of the conclusions may be related to the small number of selected patients and need to expand the sample size for further research [6]. Kreuzeder et al. found through research that concurrent radiotherapy and chemotherapy aggravated the damage of alveolar type II cells and vascular endothelial cells and increases the risk of radiation-induced lung injury. Therefore, during the treatment of thoracic malignant tumors, the incidence of radiation lung injury in patients with concurrent radiotherapy and chemotherapy is higher [7]. Chen et al. found that through the meta-analysis of amifostine, amifostine can reduce the incidence of radiation pneumonitis in patients with nonsmall cell lung cancer receiving radiotherapy. However, regarding the dosage of amifostine and the long-term efficacy of the drug on patients after treatment, the impact on overall survival is unclear and needs further study [8].

Through the use of multislice spiral CT for lung density determination of 96 patients with confirmed radiation injury and 80 medical examiners, retrospective analysis combined with literature data discusses the correlation between radiation-induced lung injury and changes in lung density.

## 2. Experimental Analysis

### 2.1. Materials and Methods

Collect 96 cases of different types of radiation-induced lung injury in our hospital from January 2003 to August 2005; among them, 43 cases were male and 53 cases were female, aged 42–88 years old. Among 96 cases, there were 38 cases of breast cancer, 32 cases of lung cancer, 10 cases of esophageal cancer, 12 cases of lymphoma, and 4 cases of mediastinal malignant tumors. All cases were confirmed by surgery or lymph node biopsy. The CT manifestations of radiation injury are classified according to the location and time of radiation: 15 cases of type 1 flaky exudation, 25 cases of type 2 patch consolidation, 25 cases of type 3 gas insufficiency, and 31 cases of type 4 chronic fibrosis. There were 80 physically fit patients, including 45 males and 35 females, aged 28–78 years old. This group of cases is not accompanied by other lung diseases (such as emphysema, bacterial pneumonia, and connective tissue disease).

### 2.2. Method

The CT scan adopts the SOMATOM Sensation 16-row spiral CT type produced by German Siemens. Lung density determination method: before the scan, train the examinee to correctly master calm breathing and use the Pulmo automatic evaluation software to set the 3 standard levels to scan under the average breathing state, that is, the pneumoconiosis level, the hilar level, and the diaphragm level. After completing the scan, use the evaluation software to draw the contours of the measured lung fields in turn for each layer of lung fields. Calculate the average CT value of 50% vital capacity. The average CT value of the 3 levels represents the average lung density of the upper, middle, and lower lung fields; the automatic software calculates the mean value of the whole lung field density (mean, ME) based on the average lung density at 3 levels, and mark this average value in the coordinates of the normal lung density range of normal people of all ages provided by Siemens; this shows that the patient's average lung density is still lower or higher than the normal average in the normal range. We divided the measured lung density CT values into 3 groups [9]: group 1: −1020–850 HU; group 2: −840–700 HU; group 3: −690–550 HU. The computer draws a pixel distribution curve according to the frequency of each pixel value in each range. All subjects underwent lung thin-layer scan. Scanning conditions: 120 kV, 120 mAs, lung window width 1200, and window level −600. The layer thickness is 3 mm, and the layer spacing is 5 mm [10–13].

### 2.3. Index Judgment

During the inspection of all research subjects, all medical staff need to pay attention to the examination status and physical signs and make records. Test five types of emphysema lung density and determine the relationship between disease type and lung density. All the data involved in this research are sorted out and analyzed by dedicated personnel [14].

### 2.4. Statistical Methods

For 4 groups of different types of radiation lung injury, the correlation test is performed based on the measured lung density. The *X*^2^ test was performed on lung density measurement for various types of radiation-induced lung injury and normal physical examination groups [15].

## 3. Results

Of the 96 different types of radiation lung injury examined, among the 25 cases of pulmonary insufficiency and 31 cases of chronic pulmonary fibrosis, there are 18 cases and 25 cases that have an average lung density higher than normal, between −750 and −620 HU. However, the average lung density values in the flaky exudative type and patch consolidation type are basically within the normal range, between −750 and −850 HU. In 80 cases in the normal physical examination group, the average lung density of 68 cases was normal, the average lung density of 12 cases was abnormal in varying degrees, compared with different types of radiation injury groups, the average lung density was 53 cases, and 43 cases were abnormal (*X*^2^ = 56.718, *P* < 0.001). The lung density difference between the radiation-induced lung injury group and the normal group was very significant (*X*^2^ = 18.027, *P* < 0.001), as given Table 1.

After comparing the two groups of subjects who underwent spiral CT examination, respiratory gas VD, and inspiratory gas MLD index, there are significant differences between the two sets of data, and it is statistically significant (*P* < 0.05).  Table 2 provides more details.

After analysis, it was found that 12 out of 30 emphysema patients had normal lung density, 18 patients had abnormal lung density, and the comparison between the two groups showed statistical significance (*P* < 0.05).  Table 3 provides more details.

**Figure 1:**
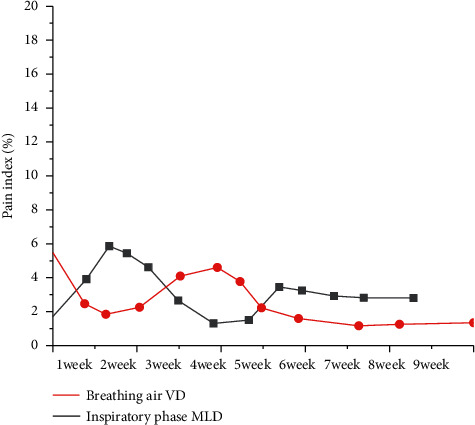
The difference of indices such as breathing gas VD and inspiratory gas MLD.

During the diagnosis process, the average lung density detection index will be affected by the patient's breathing amplitude; however, it only takes 8 seconds to scan the entire lung with 64-slice spiral CT, and the scan can be completed in one breath hold of the patient, fundamentally reducing the influence of the patient's breath on the average lung density index. There are significant differences in indices such as breathing air VD and inspiratory MLD after examination between the selected two groups of subjects, and it is statistically significant (*P* < 0.05), as shown in Figure 1. At the same time, 18 patients with emphysema have abnormal lung density [16]. The results show the importance of spiral CT lung density measurement in the diagnosis of emphysema disease. In summary, clinical emphysema disease can be diagnosed by spiral CT examination; this diagnostic method can accurately detect the lung density of patients with emphysema; in addition, the type of emphysema can be inferred based on the detected lung density, which is effective for the diagnosis and characterization of emphysema diseases.

## 4. Discussion

### 4.1. Pathogenesis

Radiation lung injury is a comorbidity that may occur in radiotherapy of thoracic tumors. Research in recent years has shown that there are two theories of radiation lung injury, one is the traditional radiation pneumonia theory. It is believed that irradiation is mainly caused by the production of local cytokines in the irradiation field, leading to fibrosis; The second is the spread of radiation pneumonia theory. It is mainly believed that it may be due to immune-mediated double lymphocytic alveolitis and local radiation field reactions. In the acute phase of the pathological process, alveolar epithelial cells and capillary endothelial cells swell and fall off progressive organization [17]. The software quantitatively analyzes CT values of different types of radiation-induced lung injury. This can not only indicate the damaged area of lung tissue in shape, range, location, radiation dose, and can also be quantified but also objectively evaluate lung density and achieve the unity of form and function.

#### 4.1.1. Cell Damage Theory

Alveolar cells, especially alveolar type II cells, are very sensitive to radiation; when exposed to radiation, its synthesis and secretion of surface active substances decrease, leading to a decrease in lung compliance; if this damage exceeds a certain level, it may cause morphological changes; in severe cases, alveolar collapse may occur, resulting in hypoxia and breathing difficulties; At the same time, vascular endothelial cells are damaged by radiation, which will cause changes in the permeability of lung microvascular, causes pulmonary ventilation and blood flow exchange barriers, aggravates alveolar type II cell damage, and further accelerates lung radiation damage [18].

#### 4.1.2. Cytokine Theory

The cytokine theory is a mainstream theory in the research of radiation lung injury; it is also a hot spot of current research, and the cytokines that cause radiation lung injury mainly include tumor necrosis factor-*α* (TNF − *α*), interleukin-6 (IL-6), transforming growth factor (TGF − *β*_1_), and so on. TNF − *α* is a potent proinflammatory cytokine mainly produced by activated monocytes or macrophages, by increasing the permeability of the capillaries in the lungs. TNF − *α* causes neutrophils and other inflammatory cells to undergo a series of inflammatory reactions under their chemotaxis. Found in the study, the TNF − *α* level can predict the occurrence and outcome of pneumonia; therefore, TNF − *α* plays a key role in the occurrence and development of radiation-induced lung injury. In the case of radioactive lung injury, alveolar macrophages can release synthetic TGF − *β*_1_ normal fibroblast phenotype transformation, thus fibroblasts' constant division, proliferation, differentiation, and maturation, and synthesis of collagen, the abnormal pulmonary interstitial collagen ingredients increased, and thus promoted the occurrence of pulmonary fibrosis; at the same time, TGF − *β*_1_ can also accelerate lung radiation injury by stimulating monocytes and inflammatory cells in the area of lung injury to release cytokines such as TNF − *α* and TNF − *β*_1_. TGF − *β*_1_ has become a recognized cytokine to predict radiation pneumonia.

#### 4.1.3. Genetic Theory

Through clinical observation, it is found that there are obvious individual differences in the occurrence of radiation-induced lung injury; therefore, the occurrence of radiation-induced lung injury may also be related to genetic factors. There are a large number of SNPs in the human genome, that is, the DNA sequence polymorphism caused by the variation of a single nucleotide, and this genetic genomic polymorphism is the genetic basis that causes differences in human diseases and different responses to treatment [19].

### 4.2. Lung Density Indicators Commonly Used in Quantitative CT Lung Density Research and Their Influencing Factors

The average lung density is affected by the patient's physique, scanning layer thickness, respiration amplitude, and reconstruction parameters and other factors. Especially, the inhalation range is the main factor. Kalender uses a breathing gate control device to accurately control breathing under the premise of 20–80% VC, the average lung density difference is close to 100 HU, and on average, there is a 16 HU lung density change for every 10% VC difference in inspiration. 16-slice spiral CT scans the whole lung in only 8–10 s; all patients can complete the scan in a certain breath hold, including those with severely reduced lung function; therefore, the influence of respiration on data determination is minimized. Our determination of lung density is a unified test under the state of 50% VC, 96 patients with radiation-induced lung injury, and all 80 normal subjects were scanned under calm breath hold. Try to avoid the difference in average lung density due to different lung capacities [20]. Therefore, the lung density measured in a stable calm breathing state is reliable and can reflect the true lung density at 50%VC. In addition, Pulmo lung quantitative software can automatically distinguish lung tissue from other tissues in the image for quantitative analysis, which greatly improves the speed and accuracy of calculation compared with the previous software and manual evaluation methods.

### 4.3. Determination and Analysis of Lung Density in Different Types of Lung Radiation Injury

We refer to Libshitz et al. that according to the degree of radiation damage to the lungs, according to the radiation dose of the anatomical part of the lung destruction area, and according to the classification of radiation pneumonia, it is divided into 4 types. Type 1 (flaky exudation type): it is more common within 4 weeks to 4.5 months after the end of radiotherapy. On the CT film, there are flaky, thin, uniform cloud-like fuzzy shadows in the radiation field. There is only a little exudation due to the lesion, and the range is small. The lung density of 15 patients with type 1 in this group is within the normal range of −700 to −820 HU. Type 2 (patch real variant): seen 25 days to 1 year after radiotherapy. The lesions can extend beyond the consolidation in the radiation field, with higher density and patch-like morphology. Some of the edges are star-shaped, and air shadows are rarely seen in the consolidation shadows, and there is traction with the surrounding pleura. In this group of 25 cases, the CT value of lung density measured between −750 and 820 HU is in the normal range. Type 3 (imperfect gas type): seen within 11 weeks to 5 years after radiotherapy. CT manifests as long strips of shadows in the radiation field, but beyond the lung segment, and in the distribution of the lung lobes, the edges are neat, and the internal observations mostly occur under the pleura, next to the lobular septum, and around the blood vessels and bronchus, the typical manifestation is a limited low-density area around the lung, often accompanied by pulmonary bullae of varying sizes. The CT value of lung density is between −850 and −950 HU. Type 4 (chronic fibrosis type): seen in 5.8–8 years. CT manifests itself in the radiation field, and there is often a margin of tax and profit between the normal lung and the radiation field, the “knife-cut edge effect” appears, the lung volume is further reduced, and the internal bronchi dilates, the number of peripheral fibrous cords increased, the interlobular septa thickened, and the bronchi and hilar machine mediastinum were stretched and displaced. The CT value of lung density is between −870 and −920 HU.

Through the measurement of CT lung density of the above types of radiation lung injury, we believe that the abnormal increase in CT values of radiation-induced lung injury may be related to the following factors. (1) Exposure dose: the total radiation dose is the most important factor, radiation doses of type 3 and type 4 patients in this document are basically >65–70 Gy and <50–55 Gy, which rarely cause radiation damage to the lungs. (2) Irradiation site: whether it is lung cancer, esophageal cancer, or breast cancer, as long as the irradiation field crosses the leaves, the pleura that affects the upper and lower mediastinum is extensively fibrotic and thickened, if the traction is significantly increased, the lung tissue volume on the side of the irradiation field can be reduced, and the CT lung density threshold of radiation dose can be increased. (3) In these data, 38 cases of breast cancer had radioactive lung injury and 35 cases of abnormal lung CT values; in 32 cases of lung cancer, radioactive lung injury caused 18 cases of abnormal lung CT values. We think that in this and radical breast cancer surgery, all pectoralis minor muscles are removed, so the risk of radiation-induced lung injury is greater than that of lung cancer. (4) Types 1-2 radiation lung injury is mostly in the early stage of radiation lung injury, and the exudation area is small, the range of involvement is mostly located at the apex of one side of the lung, and the entire volume of the lung is unaffected, so the lung CT value measurement is mostly within the normal range.

## 5. Conclusion

Clinically, emphysema is a common disease among chronic respiratory diseases, which is mostly caused by repeated bronchial asthma and chronic bronchitis, resulting in overinflating of the lungs and partial expansion of the distal end of the terminal bronchioles and the damage of lung function with the destruction of lung tissue. In the diagnosis process, the average lung density index will be affected by the patient's breathing amplitude, but it only takes 8 s at most to scan the whole lung with 64-slice spiral CT. The scanning can be completed in one breath hold, which fundamentally reduces the influence of the patient's breathing on the average lung density index. There were significant differences in respiratory VD and inspiratory MLD indices between the two groups after examination, with statistical significance (*P* < 0.05), and a total of 18 patients with emphysema had abnormal lung density. In conclusion, clinical emphysema disease can be confirmed by spiral CT examination. This diagnostic method can accurately detect the lung density of patients with emphysema and deduce the type of emphysema according to the detected lung density, which is effective in the diagnosis and characterization of emphysema disease.

## Figures and Tables

**Table 1:** Lung density measurement results of the radiation-induced lung injury group and physical examination group.

Damage type	Normal lung density group	Abnormal lung density group
Sheet exudation type	15	0
Patch reality type	25	0
Aerated incomplete type	7	18
Chronic fibrosis type	68	25
TiJianZu	6	22
Combined meter	121	55

**Table 2:** Comparison of the respiratory air VD and inspiratory MLD index of the two groups of subjects.

Group	Whole lung VD	The whole lung MLD	Lung on MLD	In the lung MLD	The lung MLD
The team	93.6 ± 37.3	−918.6 ± 40.1	−944.9 ± 32.9	−893.4 ± 31.6	−921.3 ± 29.7
The control group	202.4 ± 39.1	−761.2 ± 49.8	−811.9 ± 38.5	−775.7 ± 40.3	−734.7 ± 43.4

**Table 3:** Analysis of the measurement status of different types of average lung density in patients with emphysema.

Group	Emphysema type				
	Single lung bullae type	Lobule center type	By the interval type	Irregular shape	Central lobule type
Normal	2 (100.0)	3 (100.0)	2 (25.0)	4 (33.3)	1 (20.0)
Abnormal	0 (0.0)	0 (0.0)	6 (75.0)	8 (66.7)	4 (80.0)

## Data Availability

The data used to support the findings of this study are available from the corresponding author upon request.
